# Osteopetrotic induced pluripotent stem cells derived from patients with different disease-associated mutations by non-integrating reprogramming methods

**DOI:** 10.1186/s13287-019-1316-8

**Published:** 2019-07-17

**Authors:** Fatma Visal Okur, İnci Cevher, Cansu Özdemir, Çetin Kocaefe, Duygu Uçkan Çetinkaya

**Affiliations:** 1 Hacettepe University, Center for Stem Cell Research and Development PEDI-STEM, Ankara, Turkey; 2 Division of Pediatric Hematology and Bone Marrow Transplantation Unit, Department of Pediatrics, Faculty of Medicine, Hacettepe University, Ankara, Turkey; 3 Department of Medical Biology, Hacettepe University School of Medicine, Ankara, Turkey

**Keywords:** Osteopetrosis, Sendai virus, Episomal vector, Mesenchymal stromal cells, Induced pluripotent stem cells, Reprogramming

## Abstract

**Background:**

Autosomal recessive osteopetrosis is a genetically and phenotypically heterogeneous disease, caused by defects in osteoclast formation and function. The only available treatment is allogeneic stem cell transplantation that has still high morbidity and mortality. The goal of the present study was to generate iPSCs from bone marrow-derived MSCs of osteopetrosis patients with three most common mutations by using two different integration-free gene transfer methods and compare their efficiencies. The secondary objective was to select the most appropriate integration-free production method for our institutional iPSC bank using this rare disease as a prototype.

**Methods:**

Two different integration-free gene transfer methods (episomal and Sendai viral vectors) were tested and compared on the same set of patient samples exhibiting three different mutations associated with osteopetrosis. Generated iPSCs were characterized by standard assays, including immunophenotyping, immunocytochemistry, RT-PCR, embryoid body, and teratoma assays. Karyotype analyses were performed to evaluate genetic stability.

**Results:**

iPSC lines exhibiting typical ESC-like colony morphology were shown to express pluripotency markers by immunofluorescence staining. Over 90% of the cells were found positive for SSEA-4 and OCT3/4 and negative/weak positive for CD29 by flow cytometry. Immunohistochemical staining of teratoma and spontaneously differentiated embryoid body sections confirmed their trilineage differentiation potential. All iPSC lines expressed pluripotency-related genes. Karyotype analyses were found normal. Direct sequencing of PCR-amplified DNA showed that disease-related mutations were retained in the patient-specific iPSCs.

**Conclusion:**

Generation of iPSC using SeV and episomal DNA vectors have several advantages over other methods like the ease of production, reliability, high efficiency, and safety, which is required for translational research. Furthermore, owing to the pluripotency and self-renewal capacity, patient-specific iPSCs seem to be ideal cell source for the modeling of a rare genetic bone disease like osteopetrosis to identify osteoclast defects, leading to clinical heterogeneity in osteopetrosis patients, especially among those with different mutations in the same gene.

**Electronic supplementary material:**

The online version of this article (10.1186/s13287-019-1316-8) contains supplementary material, which is available to authorized users.

## Highlights

- Malignant infantile osteopetrosis (MIOP) is a rare genetic bone disease of childhood with no available treatment except allogeneic hematopoietic stem cell transplantations.
- Since MIOP is phenotypically and genotypically heterogeneous disease, there is still a great need for new research tools to identify the factors leading to defective osteoclastogenesis and its impact on bone metabolism and establishment of bone marrow hematopoietic niche.
- Patient-derived iPSCs carrying the disease-causing mutation would be a valuable research tool to study disease pathogenesis within the patient’s own genomic background, compared to animal models.
- Clinically relevant patient-specific iPSCs derived by integration-free reprogramming methods, like SeV and episomal vectors, will pave the way for disease modeling and discovery of new therapeutic targets, which will be of significant value to researchers and clinicians.

## Background

Osteopetrosis is a rare inherited disease characterized by increased bone mass and density resulting from defects in osteoclast formation and/or function [1, 2]. The disease is classified into two types based on the mode of transmission: autosomal dominant osteopetrosis (ADO) and autosomal recessive osteopetrosis (ARO) [3, 4]. ARO is also termed as “malignant infantile osteopetrosis” (MIOP) which presents soon after birth and often lethal unless treated accordingly [5]. A dysfunction of osteoclasts leads to bone resorption defects and increased bone density, decreased bone strength with abnormal bony overgrowth, and a bone marrow cavity insufficient to support hematopoiesis. Insufficient hematopoiesis results in bone marrow failure presented as severe cytopenias and compensatory extramedullary hematopoiesis. Other clinical manifestations resulting from bony growth are cranial nerve dysfunctions, nasal obstruction, and gross motor developmental delays. Osteoclasts are derived from hematopoietic stem cells, thus the establishment of an allogeneic graft and differentiation of functional osteoclasts after transplantation help to alleviate bone remodeling and recovery from pancytopenia as well as extramedullary hematopoiesis. Even though hematopoietic stem cell transplantation (HSCT) is the only curative treatment option, there is a high risk of graft failure and severe transplant-associated complications [1]. Disease modeling using patient-derived induced pluripotent stem cells (iPSCs) as an unlimited source of autologous cells would help to better understands the disease biology within patient’s own genomic background compared to animal models and paves the way for the development of new cell replacement therapies.

Generation of iPSCs is a milestone in stem cell research due to their unlimited maintenance capacity. iPSCs are excellent research tools to study disease modeling, drug screening, modifier gene discovery, and test novel therapeutic approaches including gene and cell replacement in a wide spectrum of inherited diseases or diseases of single gene origin or complex nature [6, 7]. Patient-specific iPSCs provide unique opportunities for both stem cell biologists and clinicians to dissect the pathogeneses and identify new alternative therapeutic strategies for rare genetic diseases by providing a virtually unlimited source of cells carrying the disease-causing mutations.

Gene delivery is crucial for the generation of iPSCs. To date, several alternatives have been developed and tested. Retroviral systems are characterized by high efficiency and reproducibility even in somatic cells that are difficult to reprogram. But they have some serious limitations such as stable expression of targeted transgenes, some of which are protooncogenes (KLF4 and c-MYC) and the possibility of insertional mutagenesis [8, 9]. For a successful translation of human iPSCs-derived products into the clinic as gene/cell therapies or other regenerative medicine applications, the safety of manufacturing strategy matters as much as its efficiency. Therefore today, integration-free methods for the generation of iPSCs are in focus [10, 11]. Somatic cell reprogramming can be achieved through various non-integrating methods such as mRNAs [12, 13] or proteins [14] or gene transfer through vectors including Sendai virus (SeV) or episomal DNA vectors [15] while avoiding the risk of insertional mutagenesis [16]. However, reprogramming efficiency using either proteins or mRNAs is still very low, and these methods require repetitive delivery of multiple proteins or mRNAs into the somatic cells. Considering the need for reprogramming technologies with higher efficiency and better safety profile for translation into the clinic, episomal vectors and Sendai viral vector which both are non-integrative are accepted to be good alternatives [17, 18]. Here, we used human bone marrow-derived mesenchymal stromal cells as the cell of origin, since they can be isolated and expanded easily from both donors and patients irrespective of their age, with a high proliferative potential. Also, they are multipotent progenitor cells, which may provide further reprogramming advantage considering the stemness of the parenteral cell [19, 20].

This study aims to generate iPSCs from bone marrow-derived MSCs of osteopetrosis patients by using two different integration-free gene transfer methods (episomal and Sendai viral vectors) and compare the efficiency of two methods in order to select the most appropriate production method. The ultimate aim of this study is use patient-derived IPS cells as a research tool to investigate the factors leading to clinical heterogeneity (as well as the discovery of modifier factors and genes) in osteopetrosis patients, especially among those with different mutations in the same gene. This might provide new insights into abnormal osteoclastogenesis and its role in the establishment of bone marrow hematopoietic stem cell niche in this rare genetic bone disease.

## Methods

### Isolation of mesenchymal stromal cells, cell culture, and characterization

Mesenchymal stromal cells (MSC) were isolated by density gradient separation from the bone marrow of two healthy donors and three patients with three different ARO associated mutations in CLCN7, TCIRG1, and SNX10 (Additional file 2: Table S1). The enriched mononuclear cell population was then cultured in complete culture medium consisting of DMEM-LG (Gibco) medium supplemented with 10% FBS, 35% MCDB (Sigma), 0.1% l-glutamine at 37 °C in a 5% CO2. Following 48 h of culture, cell attachment and morphology were evaluated. After reaching 80–90% confluency, MSCs were passaged. MSCs were characterized by flow cytometry (positive for CD29, CD44, CD73, CD90, and CD105 and negative for CD34 and CD45). The data were analyzed using BD FACS Diva Software v6.1.2, and the expression of each CD markers on the cells was calculated based on the percentage (Additional file 3: Table S2). Adipogenic and osteogenic differentiation potentials were evaluated for each established cell line. For induction of adipocyte differentiation, cells were treated with adipogenic differentiation medium (DMEM LG (Gibco) supplemented with 10% FBS (Gibco), 1 μM dexamethasone (Sigma), 60 μM indomethacin (Sigma), 500 μM 1-methyl-3-isobutylxanthine (IBMX, Sigma), and 5 μg/ml insulin (Sigma-Aldrich) for 21 days. Cells were fixed and stained with Oil Red O stain to visualize fat droplets in the cells. For osteoblastic differentiation, cells were treated with osteoblast-induction media (OB) containing DMEM-LG (Gibco) supplemented with 10% FBS (Gibco), 100 nM dexamethasone (Sigma), 10 mM beta glycerophosphate (Sigma), and 0.2 mM ascorbic acid (Sigma) for 21 days. Cells were fixed and stained with Alizarin red. The preparations of stained cells were visualized using an IX73 microscope (Olympus).

### Reprogramming of mesenchymal stromal cells

MSCs were reprogrammed by two different vector systems. A mixture of three Sendai virus-based (SeV) vectors expressing a classic set of reprogramming transgenes Oct4, Sox2, Klf4, and c-Myc (CytoTune-iPS Sendai Reprogramming Kit, A1378001, Invitrogen) (29) and an episomal vector system (Epi5) containing an optimized mixture of three oriP/EBNA-1(Epstein-Barr nuclear antigen-1)-based episomal plasmids (30). The latter approach uses Oct4, Sox2, Lin28, L-Myc, and Klf4 as the transgenes for reprogramming and also utilize two additional plasmids expressing mp53DD (dominant-negative mutation of p53 protein) and EBNA1 to facilitate episomal plasmid DNA replication in dividing cells to enhance reprogramming efficiency (Epi5, Episomal iPSC Reprogramming Kit, A15960, Invitrogen) [21, 22].

#### SeV reprogramming

One day prior to transduction, early passage (P3-P4) MSCs were harvested with Accutase, plated in a 6-well plate coated with 0.1% gelatine at 1 × 105 cell/ well density and maintained in MSC growth medium (10% FBS, 1% l-glutamine (2 mM), low-glucose (3.5 g/dl) DMEM). Next day, 80–85% confluent MSCs were transduced with SeV vectors at MOI of 5:3:3 in 2 ml fresh MSC medium for 24 h. At day 5, the cells were harvested with Accutase, counted and seeded in a Matrigel-coated 10 cm dish at 1 × 105 cells/well confluence and switched into TeSRTM-E7TM reprogramming medium (Stemcell Technologies). Cells were maintained in TeSRTM-E8TM (Stem Cell Technologies) medium starting from day 14. IPS colonies that started to appear around day 18 were harvested with manual microdissection method, transferred into Matrigel-coated dishes, and were expanded using EDTA clump passaging approach [23].

#### Episomal reprogramming

Early passage (P3-P4) MSCs at 80–85% confluence were harvested with Accutase, and 1 × 106 cells were re-suspended in nucleofector solution supplied in the P1 Primary Cell 4D-Nucleofector® X Kit L (Lonza). One microliters of an optimized mixture of Epi5 TM reprogramming plasmids (Oct4, Sox2, Klf4, L-Myc, and Lin28) and 1 μl of an optimized mixture of mP53DD and EBNA1 plasmids were added to the cell suspensions. pMax-GFP was used as a transfection control plasmid. The cell suspension was transfected by using program FF-104 on a 4D-NucleofectorTM X Unit. In order to assess the transfection efficiency, GFP expression on transfected MSC cells was checked by both immunofluorescence microscopy (Olympus-IX73) and flow cytometry 24 h later. Following the identification of the IPS colonies, cells were harvested and maintained as described above.

Reprogramming efficiencies of both methods were determined by alkaline phosphatase (AP) staining of the whole plate (described below) at around day 24–28 post-transduction/post-transfection when colonies were ready for picking. The reprogramming efficiency was defined as the ratio of the number of colonies positive for alkaline phosphatase activity to the total number of cells used in the reprogramming experiment.

### Characterization of iPSC lines

Besides colony morphology and alkaline phosphatase activity, immunofluorescence staining, flow cytometry, and karyotype analyses were performed for the characterization of selected IPS lines. Expression of pluripotency genes and detection of residual SeV and episomal plasmid sequences were evaluated in reprogrammed IPS cells using real-time PCR and conventional PCR. Embryoid body formation and teratoma assays were performed to assess pluripotency in vitro and in vivo. As per each sample, three IPS lines were characterized.

Alkaline phosphatase activity of iPSCs was determined by staining with Blue AP Staining Kit vector labs SK-530. Immunofluorescence staining was done using PSC 4-Marker Immunocytochemistry Kit (Life Technologies). In brief, after fixation and permeabilization, IPSC colonies were first incubated with primary antibodies (OCT4, SSEA4, SOX2, and TRA1–60) for 3 h, and then with conjugated secondary antibodies for 1 h at RT. Following staining with DAPI, samples were examined on a fluorescence microscope (Olympus- IX73).

Expression of pluripotency markers on iPS cells was evaluated with flow cytometry (FACS Aria and Navios EX Beckman Coulter). iPSC colonies were harvested with Accutase, and single cell suspensions were stained using antibodies against SSEA4, OCT4, and CD29 (Additional file 3: Table S2). Gates on flow cytometry were defined according to isotype controls.

Genetic stability of iPSC cell lines was evaluated by standard Giemsa-banded karyotype analysis. Verification of the retained mutations was assessed by sequencing of the genomic locus.

The mRNA expression levels of the pluripotency genes (Endo-Oct4, Endo-Sox2, Nanog, c-Myc, Klf-4, Utf-1, Dnmt3b, Tert-1, Rex-1, CDH-1,) were analyzed by reverse transcriptase coupled quantitative real-time PCR (qRT-PCR). Total RNA isolation was conducted by Promega, ReliaPrep™ RNA Cell Miniprep System (Cat no: Z6012). qPCR studies were performed using ThermoFisher Maxima SYBER Green/ROX qPCR master mix on a Rotorgene 6000 (Corbett Life Science, Australia) fluorometric PCR instrument. Gene expression was normalized to the expressions of β-actin and TFIID as reported previously [24]. The losses of Sendai virus genome (SeV genome sequence targeted) and episomal DNA (OriP sequence targeted) were assessed by end-point PCR using Promega GoTaq® DNA polymerase (Additional file 3: Table S2). Agarose gel electrophoresis results of end-point PCR analysis of cDNA expression were presented.

In vitro trilineage differentiation potential of iPSC lines was evaluated with embryoid body (EB) assay. iPSC colonies were harvested using Accutase, single-cell suspensions were prepared at passage 15–20, and cells were seeded onto AggreWellTM800 plates (StemCell Technologies). Following a 24-h suspension culture, EBs were harvested and transferred to ultra-low attachment plates in STEMdiffTM APELTM 2 Medium (StemCell Technologies) and kept in culture for spontaneous differentiation. Differentiated EBs were stained with hematoxylin and eosin. Microsections of paraffin-embedded EBs were also stained with antibodies against OCT4, vimentin, and synaptophysin.

In vivo trilineage differentiation potential of iPSCs was demonstrated with teratoma assay. iPSCs (passages 15–20) in six-well plates were dissociated manually. iPSCs were injected subcutaneously into 3-month-old female Blb/C-Nude mice. Around 8-week post-injection terotoma formation was observed, mice were sacrificed and teratoma was dissected. Immunohistochemical stains for hematoxylin and eosin as well as Mallory trichrome and Toluidine Blue were performed for the evaluation of lineage-specific differentiation.

#### Statistical analysis

*P* values were calculated using chi-square test and method-specific efficiencies were provided as geometric means ± SEM. Arbitrary gene expression levels were achieved by normalizing the gene of interest to the geometrical mean expressions of reference genes as described previously [24]. The arbitrary gene expression was further normalized to the mean expression of control samples to achieve fold change values. Analysis of variance was conducted on the replicate values of experiment groups, and the groups satisfying statistical significance were indicated where appropriate (*). The data was analyzed using R statistical package (version 3.5.1) using BRB Array Tools interface (version 4.6.0).

## Results

### Bone marrow-derived mesenchymal stromal cell culture and characterization

BM-MSCs were characterized by morphology, immunophenotyping, and evaluation of differentiation potential. Mycoplasma testing proved that all MSC lines were free from contamination. Flow cytometry analyses showed that all tested MSCs expressed specific surface markers of mesenchymal stromal cells, CD29, CD44, CD73, CD90, and CD105, and they were found negative for CD34 and CD45. Mesodermal lineage differentiation potential of BM-MSCs was assessed by induction of adipogenic and osteogenic differentiation (Additional file 1: Figure S1).

### Generation, expansion, and characterization of iPSC lines from BM-MSCs of osteopetrosis patients

We performed both SeV- and Epi5-mediated inductions from three patients who had disease-associated mutations in three different genes (TCIRG1, SNX10, and CLCN7) representing the diverse genetic heterogeneity of osteopetrosis phenotype and two healthy donors, under the same culture conditions. Three IPS lines per sample for each method were selected and characterized using the standard pluripotency assays. Reprogramming efficiencies were calculated and loss of SeV genome and Episomal plasmid were tested to understand the time-frame of the loss of episomal agents along with iPSC expansion. Finally, verification of the mutations was assured for all IPSC lines derived from osteopetrosis patients.

Transfection efficiency of episomal plasmids in BM-MSCs was evaluated by EGFP expression and was found to be 72% ± 3.46. The first ES- like colonies were observed at around day 18 post-transduction with SeV and around day 21 post-transfection with episomal plasmids. For each patient’s line, 8–10 iPSC colonies were picked manually and further expanded in culture. Control plates were stained with alkaline phosphatase to identify iPSC colonies, and the mean efficiencies of successful reprogramming experiments of osteopetrotic BM-MSCs were found as 0.3 ± 0.11% for SeV and 0.1 ± 0.02% for Epi5 method, higher than those of the control (0.14 ± 0.01% for SeV and 0.07% for Epi5).

**Fig. 1:**
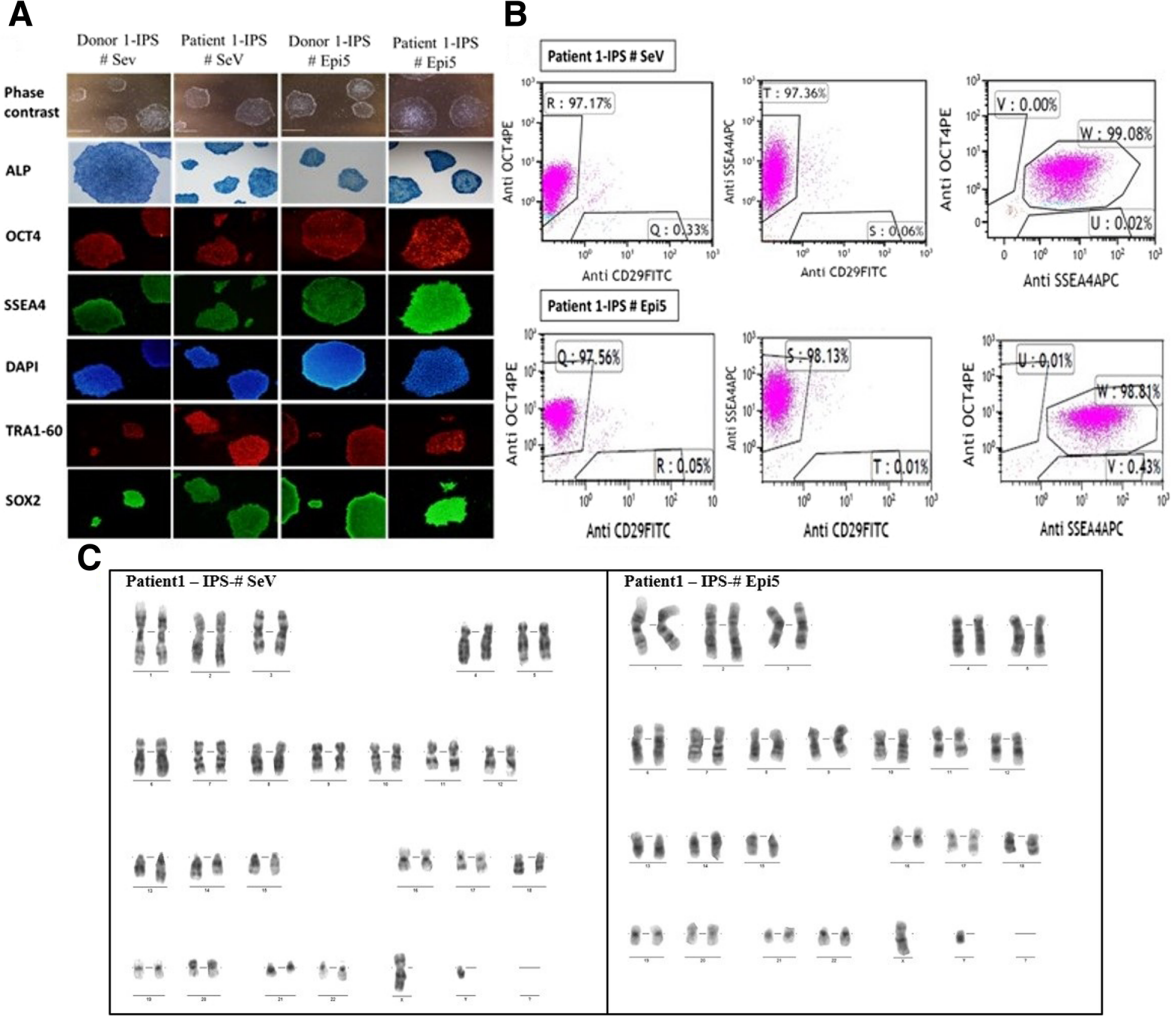
Characterization of established iPSC lines derived from patient and donor BM-MSCs using SeV and Epi5. **a** Representative images of iPSC colonies with a typical embryonic stem cell colony morphology and alkaline phosphatase (ALP) activity. Immunostaining with pluripotency markers and DAPI. **b** Flow cytometry analysis with pluripotency markers OCT4, SSEA4 and MSC marker CD29 as control. **c** Karyotype analysis of iPSC lines for genetic stability performed at P20

**Fig. 2:**
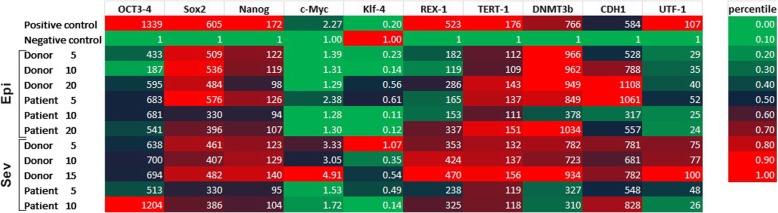
Relative expression of pluripotency genes in established iPSC lines**.** Relative arbitrary expression values are normalized to the negative control sample and indicated as fold change. Endogenous OCT4 and SOX2 expressions were analyzed differentially to demarcate any expression originating from the transgenes. The cumulative expressions were given for all the other genes. The expressions of pluripotency genes were detectable in all of the tested lines for all time-points, with no significant variations between the lines (*n* = 47 iPSC lines, three lines from each sample at different time points; P5-P20)

All hiPSC lines derived from osteopetrosis patients expressed alkaline phosphatase and other stemness markers (SSEA4, OCT3/4, TRA1-60, SOX2) as revealed by immunofluorescence staining, flow cytometry (SSEA4, OCT3/4) (Fig. 1a), and qRT-PCR (Fig. 2). Flow cytometry analyses demonstrated that more than 90% of SeV- and Epi5-derived iPSCs were positive for SSEA4 (SeV 97.21% ± 0.69; Epi5 99% ± 0.11 *P* > 0.05) and OCT3/4 positive (SeV 94.09% ± 0.63; Epi5 94% ± 1.07 *P* > 0.05), while they were either negatively or weakly positive for CD29 (SeV 1.6% ± 0.36; Epi5 2.7% ± 0.81 *P* > 0.05) (Fig. 1b). Karyotype analyses were found normal for all iPSC lines (Fig. 1c). Mutation verification showed that disease-related mutations were retained in the iPSCs.

The analyses of pluripotency gene expression panel consisted of ten different target mRNA. Out of the four transgenes (OSKM factors) that were utilized for reprogramming, OCT4 and SOX2 were differentially assessed to delineate endogeneous versus exogenic (transgene) expression, since the timing and the levels of OCT4 and SOX2 expression are important indicators of reprogramming efficiency [25]. The cumulative expressions of other investigated transcripts (KLF4, c-MYC, NANOG, REX1, TERT1, DNMT3b, CDH1, UTF1) were presented (Fig. 2).

**Fig. 3:**
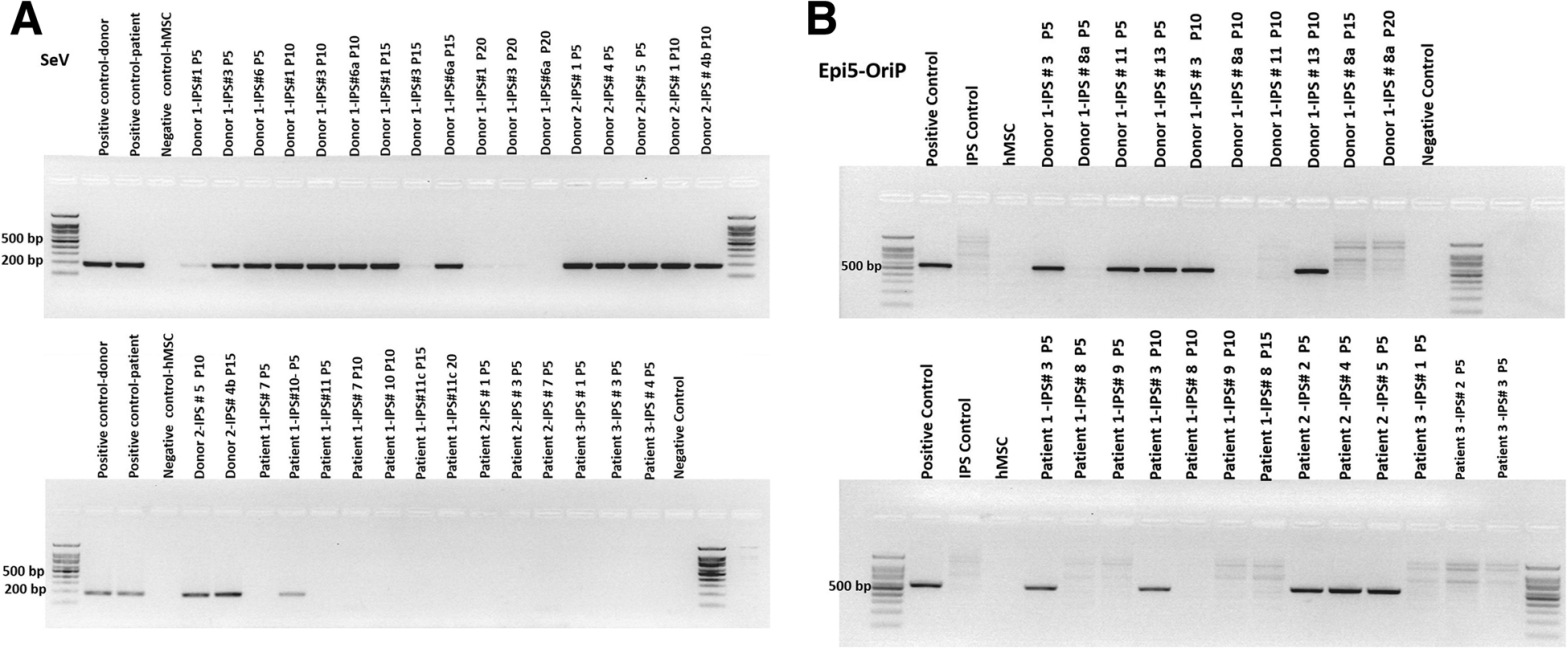
**a**, **b** Retention of reprogramming agents. SeV and Epi5 derived IPS lines show different dynamics of the loss of viral genome (**a**) and episomal plasmid (**b**)

The pluripotency gene expressions were evaluated at different time points (passages 5–20) for each established iPSC lines. Results revealed that the induction of pluripotency was already achieved at P5 and none of the investigated genes exhibited any significant time-course variance along the observed period for IPSC lines obtained with both methods. The major observed difference is the absence of the expression of investigated genes in a negative control sample (primary BM-MSC) excluding KLF4 [26], (Fig. 2). In this regard, the presence of the expression of these genes discriminate samples from negative controls in an on/off manner. Next, we tested how quickly the exogenous reprogramming agents were lost during iPSC expansion. We observed a passage-dependent decrease in both SeV RNA and episomal DNA levels in all iPSC lines, but an accelerated loss of SeV RNA in all the patient-iPSC lines and all SeV patients IPS lines were negative by P5. The retention of the episomal genome in both Epi5 and SeV induced IPS colonies derived from the same control is probably sample-dependent and may be related to epigenetic and proliferative features of the donor BM-MSCs (Fig. 3).

**Fig. 4:**
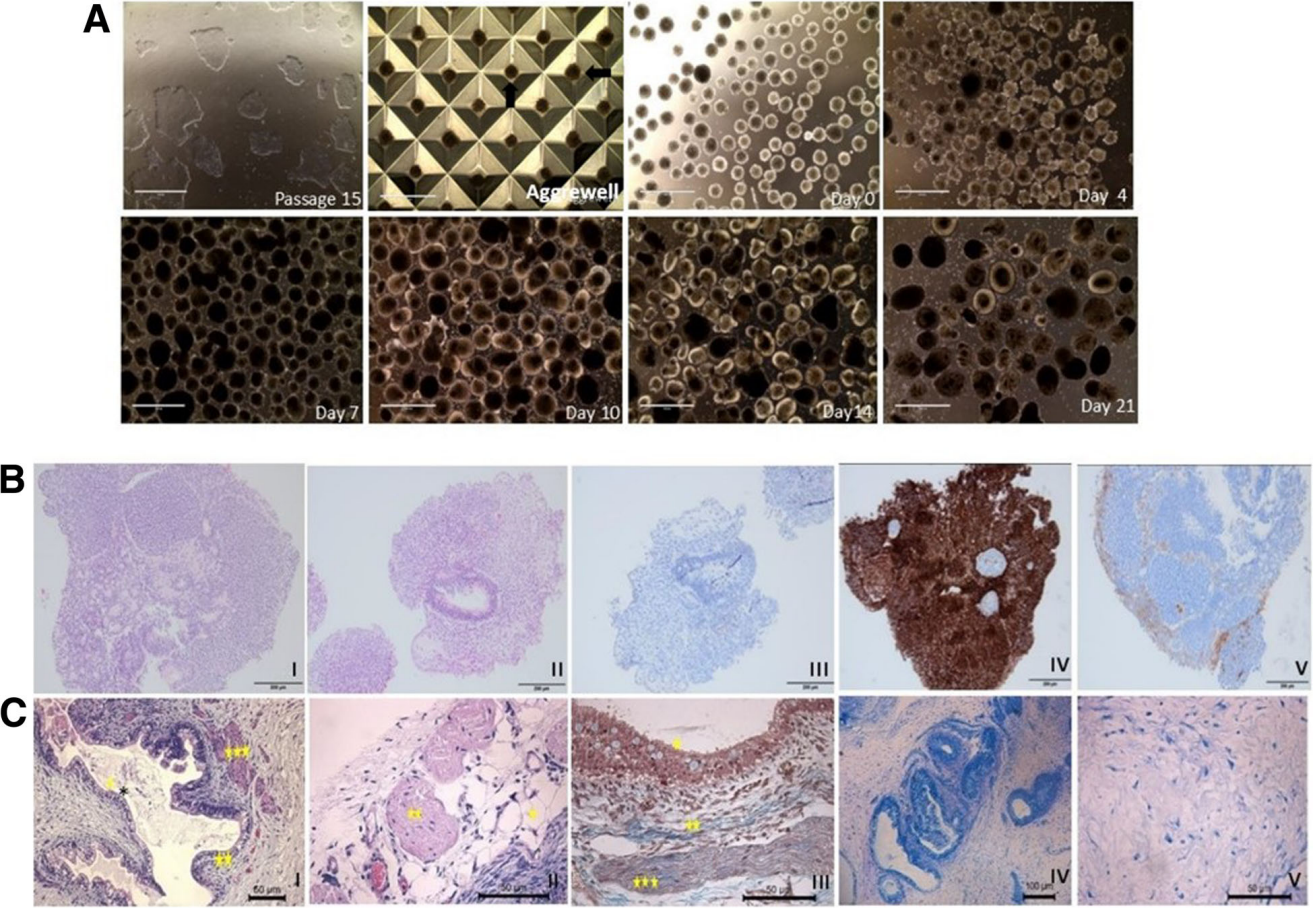
Trilineage differentiation potential of established iPSC lines of osteopetrosis patients. **a** Formation of embryoid bodies from iPSC lines in AggreWells and morphological changes observed during spontaneous differentiation (Patient 1-IPS-P15# 8a-Epi). Cell clumps within the Aggrewell pointed out with arrows. **b** H&E and immunohistochemical staining of differentiated embryoid bodies on day 21; I—mesenchymal and neuroepithelial area (H&E); ii—mesenchymal and primitive epithelial area (H&E); III—negative OCT4 staining; IV—positive vimentin staining indicating mesodermal differentiation; V—positive synaptophysin staining indicating ectodermal differentiation (Patient 1-IPS-P15# 8a-Epi). **c** H&E and immunohistochemical staining of teratoma sections. Tissue derivatives indicative of the three germ layers observed, I, III: *epithelial and goblet cells, **connective tissue, ***smooth muscle; II: *epithelial and goblet cells,**peripheral nerve; IV: connective tissue and goblet cells; V: gray matter of central nervous system (I, II H&E, III Mallory Trichrom, IV, V Toluidin) (Patient 1-IPS-P20#11c-SeV)

Finally, in vitro trilineage differentiation potential of generated hiPSC lines were evaluated with EB formation and ability of iPSC-derived EBs to form representatives of three germ layers. Uniform size- and spheroid-shape EBs were maintained in suspension culture to induce spontaneous differentiation up to 21 days before immunohistological analyses (Fig. 4a). Morphological change similar to early embryogenesis such as increase in size, 3D spheroidal appearance, development of an inner cell layer and formation of cystic areas were observed over time. Hematoxylin and eosin-stained histologic sections of spontaneously differentiated derivatives showed trilineage potential. Expression of neuroectodermal (synaptophysin) and mesodermal (vimentin) markers in the differentiated EBs, together with lack of pluripotency marker (OCT3/4) confirmed their differentiation potential (Fig. 4b).

In vivo trilineage differentiation potential of patient-specific iPSCs was demonstrated by teratoma formation assay. Histological examination of the teratomas revealed the presence of a set of representative tissues that were originated from the three embryonic germ layers, including epithelium, muscle, connective tissue, peripheral nerve, and central nervous system (Fig. 4c).

Taken together, our results demonstrate that iPSCs could be derived efficiently from osteopetrosis patients with different disease-associated mutations, using two different integration-free reprogramming methods. There was not any significant qualitative difference among the iPSC lines obtained with compared two methods.

## Discussion

Autosomal recessive osteopetrosis is a genetically and phenotypically heterogeneous disease, which is caused by defects in osteoclast formation and function. Although seven genes have been identified so far as having a role in disease pathogenesis, a study on disease-associated mutations continues to discover essential genes for osteoclast function and to better understand osteoclast biology. Use of patient-derived pluripotent stem cells could be an alternative to genetically modified cell lines or animal models for these functional studies, providing a virtually unlimited source of autologous cells carrying the disease-causing mutations. In this study, we derived and expanded iPSC lines successfully from osteopetrosis patients with mutations in TCIRG1, CLCN7 and SNX10 genes (accounting nearly 70% of all cases) using two different integration-free reprogramming methods under feeder-free culture conditions.

Both SeV and Epi5 reprogramming methods were found quite efficient and highly reliable for the generation of patient-specific iPSCs from BM-MSCs. Characterizations confirmed that the generated iPSCs expressed pluripotent stem cell markers, showed trilineage differentiation potential, displayed normal karyotype and retained the disease-associated mutations.

We evaluated the acquisition of the pluripotency state through the investigation of several pluripotency-related transcripts in iPSC lines at selected time points. Excluding some of the investigated genes, these data did not exhibit any striking variance in a time course manner. The observed variations for some of the investigated transcripts may reflect the natural fluctuation of gene expression in between samples, which may be secondary to the epigenetic state of the reprogrammed cell genome as well as the exposure to the reprogramming factors. The variant clustering of KLF4 and cMyc genes among control BM-MSCs may be related to the nature of these transcripts in BM-MSCs, but may not exhibit a similar pattern in iPSC reprogramming of a cell from any other somatic source [26]. These data show that iPSC reprogramming is an on/off phenomenon that would remain unchanged over time once the culture conditions are maintained standard, independent from the method of reprogramming. Similarly, Trevisan et al. who investigated the impact of the reprogramming methods (retrovirus, SeV, and episomal) on the quality of iPSCs identified no significant difference among the stem cell gene expression profiles of iPSCs derived with different methods [27].

Our results support the study of Daley et al. comparing three different non-integrating reprogramming methods (SeV, Episomal, and mRNA) using a number of criteria [21]. They reported the reprogramming efficiencies as 0.077% for SeV and 0.013% for Epi5. Higher efficiencies in our experiments for both methods compared to the healthy donor BM-MSCs could be attributed to primary disease-specific and/or parenteral cell-specific features that make BM-MSCs more prone to reprogramming. They observed a passage-dependent decrease in viral load over time in SeV-mediated iPSC lines which occurred at later passages, relative to episomal sequences. We also observed a passage-dependent decrease in reprogramming agents in all of the iPSC lines and complete loss at higher passages, but an accelerated loss of SeV RNA in all the patient-iPSC lines. The retention of episomal genome is probably sample-dependent and may be related to epigenetic and proliferative features of the donor BM-MSCs.

In another study evaluating the integration-free reprogramming methods for obtaining clinically relevant iPSCs, there were not any qualitative differences in iPSC lines, except reprogramming efficiencies [16]. It seems that major determinants of reprogramming success are sample-related factors, cell of origin (*proliferation and differentiation status of cell and cellular epigenetic features*), and culture conditions rather than the method itself [28]. A range of somatic cell types including neonatal fibroblasts, dental pulp cells, adipose cells, CD34+ cells from the umbilical cord, and peripheral blood mononuclear cells has been successfully reprogrammed with SeV and Episomal methods [11, 17, 29, 30]. We preferred to reprogram BM-MSC of osteopetrosis patients, because of their multipotency, ease of purification/expansion and well-established characteristics [19, 20]. As we hypothesized iPSC lines derived readily from BM-MSCs of all patients and healthy donors with high efficiency. BM-MSC-derived osteopetrotic iPSCs would also be a valuable research tool to investigate defective osteoclastogenesis and interaction between osteoclasts and hematopoietic niche, considering phenotypical heterogeneity of patients.

Both SeV and Episomal vectors allow expression of transgenes without risk of host genome modification. Reprogramming efficiency of SeV method is higher than that by other methods, especially without using any small molecules. Also, it is possible to select iPSCs that depleted viral genome using an antibody against viral HN protein expressed on surface of infected cells. Episomal plasmids, on the other hand, can be manufactured and qualified for GMP use at a lower cost. Reprogramming efficiency of episomal plasmids can be boosted further using epigenetic modifiers or other reprogramming factors. Thus, the choice of reprogramming method should be dependent on applications in which generated iPSC lines will be used such as in vitro stem cell research or clinical translation.

## Conclusion

In conclusion, we reprogrammed bone marrow-derived MSCs of osteopetrosis patients with three different osteopetrosis-associated mutations in three different genes by using two different integration-free gene transfer methods (episomal and Sendai viral vectors) and the efficiency of two methods were compared to select the most appropriate production method. There were not any method-specific differences in the expression levels/patterns of pluripotency markers and the developmental potential in generated iPSC lines.

Patient-derived iPSCs would provide limitless cell source to study osteoclast defects associated with disease-specific mutations within the context of a patient’s whole genome. This is important for the understanding of the factors leading to clinical heterogeneity in osteopetrosis patients especially among those with different mutations in the same gene. These investigations will also pave the way for identification of new therapeutic targets, testing new drugs and development of genetically modified cell therapy products.

## Additional files

Additional file 1: **Figure S1.** Characterization of BM-MSCs derived from osteopetrosis patients and the donor. A) Representative phase images of the patient and donor MSCs. B) Flow cytometry analysis with MSC-specific markers CD29, CD44, CD73, CD90, and CD105. CD34 and CD45 are negative markers for MSCs. C) Mesodermal differentiation potential of BM-MSCs. Representative images of differentiated donor-MSCs and patient-MSCs, i- adipocytes (positive for oil red O stain), ii- osteoblasts (positive for Alizarin red stain), iii- negative control cells. (PDF 213 kb)

Additional file 2: **Table S1.** Resource table. (DOCX 16 kb)

Additional file 3: **Table S2.** Reagents and primer sequences. (DOCX 17 kb)

## Data Availability

All data and materials are available in the manuscript.

## Abbreviations

ADO: Autosomal dominant osteopetrosis
AP: Alkaline phosphatase
ARO: Autosomal recessive osteopetrosis
BM-MSC: Bone marrow mesenchymal stem cell
c-MYC: c-MYC binding protein (MYCBP) pseudogene
CDH1: Cadherin 1
cDNA: Complementary DNA
CLCN7: Chloride voltage-gated channel 7
DAPI: 4′,6-Diamidio-2-phenylindole dihydrocloride
DMEM-LG: Dulbecco’s modified Eagle medium
DNA: Deoxyribonucleic acid
DNMT3B: DNA methyltransferase 3 beta
EB: Embryoid body
EBNA-1: Epstein-Barr nuclear antigen-1
EDTA: Ethylenediaminetetraacetic acid
EGFP: Enhanced green fluorescent protein
Endo-Oct4: Endogenous OCT4
Endo-Sox2: Endogenous SOX2
Epi5: Episomal Vector System
ESC: Embryonic stem cell
FACS: Fluorescence-activated sorting
FBS: Fetal bovine serum
GFP: Green fluorescence protein
GMP: Good manufacturing practice
H&E: Hematoxylin and eosin
HSCT: Hematopoietic stem cell transplantation
IBMX: 1-Methyl-3-isobutylxanthine
iPSCs: Induced pluripotent stem cells
KLF4: Kruppel like factor 4
MIOP: Malignant infantile osteopetrosis
MOI: Multiplicity of infection
MSCs: Mesenchymal stem cell
mRNA: Messenger RNA
NANOG: Nanog Homeobox
OCT3/4: Octamer-binding transcription factor 4
PCR: Polymerase chain reaction
REX1: RNA exonuclease 1 homolog pseudogene
RNA: Ribonucleic acid
RT-PCR: Real-time PCR
qPCR: Quantitative PCR
qRT-PCR: Quantitative real-time PCR
SEM: Standard error of the mean
SeV: Sendai viral vector
SNX10: Sorting nexin 10
SOX2: SRY-box 2
SSEA-4: Stage-specific embryonic antigen-4
TCIRG1: T cell immune regulator 1, ATPase H+ transporting V0 subunit a3
TERT: Telomerase reverse transcriptase
UTF1: Undifferentiated embryonic cell transcription factor 1
