# Supplementary material for: Osteopetrotic induced pluripotent stem cells derived from patients with different disease-associated mutations by non-integrating reprogramming methods
Source: Stem Cell Res Ther. 2019 Jul 17;10:211. doi: 10.1186/s13287-019-1316-8 (PMC6637500; doi:10.1186/s13287-019-1316-8)
Supplement: Supplementary file 1 — Figure S1. Characterization of BM-MSCs derived from osteopetrosis patients and the donor. A) Representative phase images of the patient and donor MSCs. B) Flow cytometry analysis with MSC-specific markers CD29, CD44, CD73, CD90, and CD105. CD34 and CD45 are negative markers for MSCs. C) Mesodermal differentiation potential of BM-MSCs. Representative images of differentiated donor-MSCs and patient-MSCs, i- adipocytes (positive for oil red O stain), ii- osteoblasts (positive for Alizarin red stain), iii- negative control cells. (PDF 213 kb) [file 13287_2019_1316_MOESM1_ESM.pdf]

Figure S1. Characterization of BM-MSCs derived from osteopetrosis patients and the donor (related to the information provided in the experimental procedures)

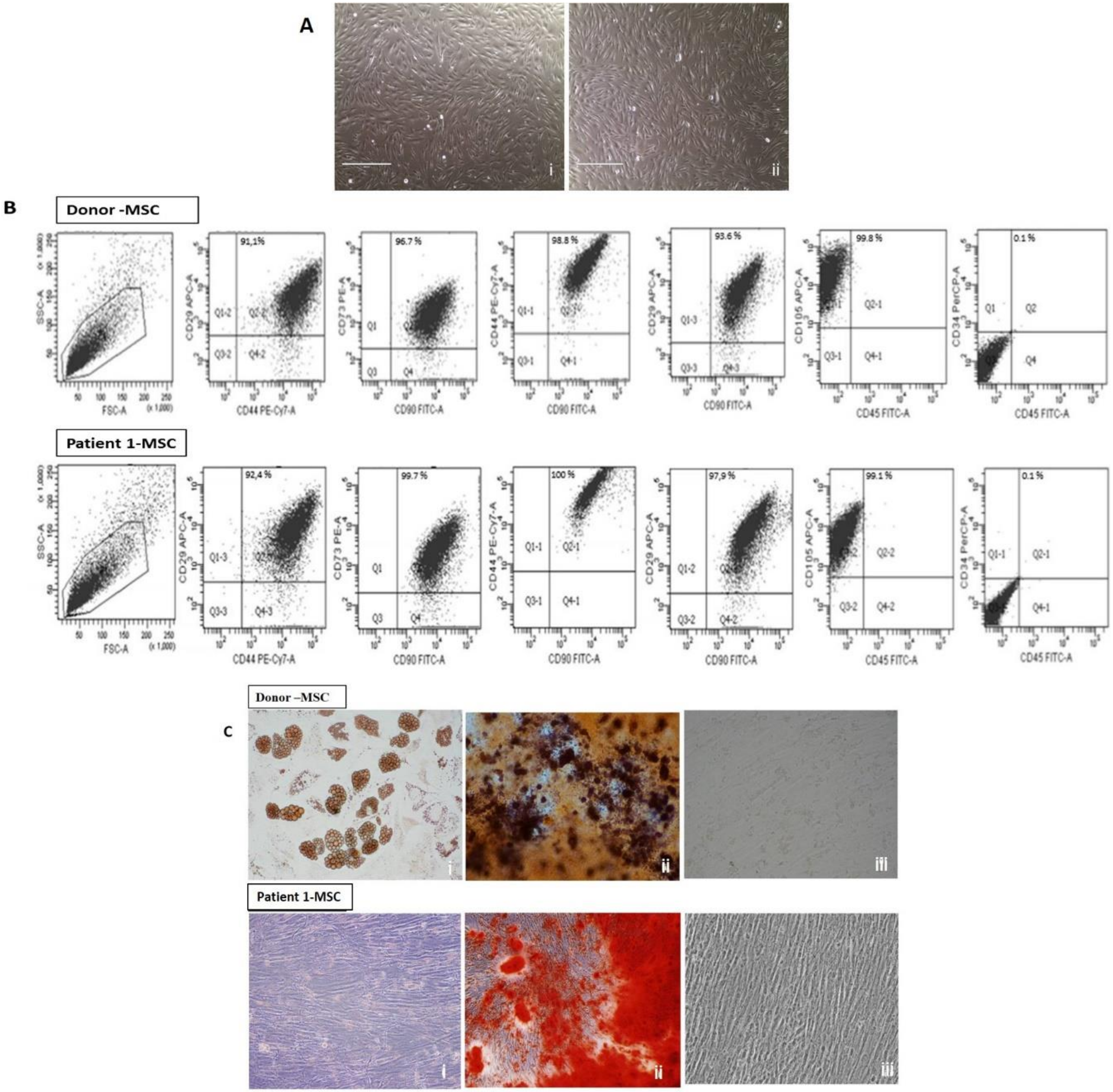

Figure SI. Characterization of BM-MSCs derived from osteopetrosis patients and the donor. A) Representative phase images of patient and donor MSCs. B) Flow cytometry analysis with MSC-spesific markers CD29, CD44, CD73, CD90 and CD105. CD34 and CD45 are negative markers for MSCs. (C) Mesodermal differentiation potential of BM-MSCs. Representative images of differentiated MSCs, i- adipocytes (differentiated cells positive for oil red O stain), ii- osteoblasts (differentiated cells positive for Alizarin red stain), iii- negative control cells.
