# Supplementary material for: Osteopetrotic induced pluripotent stem cells derived from patients with different disease-associated mutations by non-integrating reprogramming methods
Source: Stem Cell Res Ther. 2019 Jul 17;10:211. doi: 10.1186/s13287-019-1316-8 (PMC6637500; doi:10.1186/s13287-019-1316-8)
Supplement: Supplementary file 2 — Table S1. Resource table. (DOCX 16 kb) [file 13287_2019_1316_MOESM2_ESM.docx]

**Additional Table 1.** Resource table ( related to the information provided in the experimental procedures )

| Unique stem cell line identifier | Patient 1-BSG-OST14 –MSC (TCIRG1)  Patient 2-ZCD-OST10-MSC (SNX10)  Patient 3-ANÇ-OST3-MSC (CLCN7)  Donor 1 - ES-Donor-MSC  Donor 2- UB-Donor-MSC |
| --- | --- |
| Alternative name(s) of stem cell line | **Patient 1-IPS#** colony number, passage, SeV/ Epi5  **Patient 2-IPS#** colony number, passage, SeV/ Epi5  **Patient 3-IPS#** colony number, passage, SeV/ Epi5  **Donor 1 -IPS#** colony number, passage number- SeV  **Donor 2 -IPS#** colony number, passage number- SeV/ Epi5 |
| Institution | Hacettepe University Center for Stem Cell Research and Development (PEDI-STEM). Ankara, Turkey. |
| Contact information | Fatma visal okur, [fvokur@hacettepe.edu.tr](mailto:fvokur@hacettepe.edu.tr) |
| Type of cell line | iPSC |
| Origin Human | Human |
| Additional origin info Donor information | **Donor 1:** Age: 6 years old, Sex: Female, Ethnicity: Turkey  **Donor 2:** Age: 2 years old, Sex: Female, Ethnicity: Turkey |
| Additional origin info Patients information | **Patient 1:** Age: 9 monhts old, Sex: Female, Ethnicity: Turkey  **Patient 2:** Age: 15 months old, Sex: Female, Ethnicity: Turkey  **Patient 3:** Age: 7 months old,Sex: Female, Ethnicity: Turkey. |
| Cell source | Mesenchymal Stem Cell |
| Method of reprogramming | Sendai virus and Episomal vector |
| Associated disease | Autosomal recessive osteopetrosis (ARO) |
| Gene/locus | **Patient 1:** “g.2862 G>A “ mutation in T cell immune regulator 1, ATPase H+ transporting V0 subunit a3 (TCIR G1)  **Patient 2:** “g.80684_80685” insA mutation in Sorting Nexin 10 (SNX10) gene  **Patient 3:”**c.484 + 4T>C “ mutation in chloride voltage-gated channel 7 (CLCN7) gene |
| Method of modification | N/A |
| Gene correction | N/A |
| Name of transgene or resistance | N/A |
| Inducible/constitutive system | N/A |
| Date archived/stock date | N/A |
| Ethical Approvel | Written informed consents for collection, storage and use of cells for research purposes were obtained and the study is approved by the Instituonal Review Board of Hacettepe University (Study approval number: 16969557-1213). |
