# Supplementary material for: Osteopetrotic induced pluripotent stem cells derived from patients with different disease-associated mutations by non-integrating reprogramming methods
Source: Stem Cell Res Ther. 2019 Jul 17;10:211. doi: 10.1186/s13287-019-1316-8 (PMC6637500; doi:10.1186/s13287-019-1316-8)
Supplement: Supplementary file 3 — Table S2. Reagents and primer sequences. (DOCX 17 kb) [file 13287_2019_1316_MOESM3_ESM.docx]

**Additional Table 2.** Antibodies used in flow cytometry and immune flourescence staining, and primer sequences used in RT-PCR analyses (related to the information provided in the experimental procedures)

| Antibodies used for flow-cytometry | | | |
| --- | --- | --- | --- |
|  | Antibody | | Company Cat# |
| Mesenchymal stem cell | CD29 APC | | BioLegend 303008 |
| Mesenchymal stem cell | CD29-Alexa-488 | | Serotec MCA229898A488 |
| Mesenchymal stem cell | CD44 PE-Cy7 | | BD 560533 |
| Mesenchymal stem cell | CD73 PE | | BD 344014 |
| Mesenchymal stem cell | CD90 FITC | | BD 555595 |
| Mesenchymal stem cell | CD105 APC | | BD 562408 |
|  |  | |  |
| Hematopoietic stem cell | CD34 PerCP | | BD 343606 |
| Hematopoietic stem cell | CD45 FITC | | BioLegend 304006 |
| Pluripotency marker | SSEA4 APC | | R&D FAB1435A |
| Pluripotency marker | OCT4 PE | | BD 560186 |
|  | | | |
| Antibodies used for immunoflourescent | | | |
| Antibody | | | Company Cat# |
| Pluripotency marker | | anti-OCT4 (host: rabbit) | Life technologiesA24867 |
| Pluripotency marker | | anti-SSEA4 (host: mouse IgG3) | Life technologies A24866 |
| Pluripotency marker | | anti-SOX2 (host: rat) | Life technologies A24759 |
| Pluripotency marker | | anti-TRA-1-60 (host: mouse IgM) | Life technologies A24868 |
|  | |  |  |
| Secondary antibodies | | Alexa Fluor® 555 donkey anti-rabbit; for use with anti-OCT4 | Life technologies A24869 |
| Secondary antibodies | | Alexa Fluor® 488 goat anti-mouse IgG3; for use with anti-SSEA4 | Life technologies A24877 |
| Secondary antibodies | | Alexa Fluor® 488 donkey anti-rat; for use with anti-SOX2 | Life technologies A24876 |
| Secondary antibodies | | Alexa Fluor® 555 goat anti-mouse IgM; for use with anti-TRA-1-60 | Life technologies A24871 |

| **Gene Name** |  | **Primer Sequence** | **Accession Numbers** |
| --- | --- | --- | --- |
| **House Keeping Gene** | | | |
| Beta-Actin | FP | 5'-AAAATCTGGCACCACACCTTC-3’ | NM_001101.4 |
|  | RP | 5'-AGCACAGCCTGGATAGCAAC-3’ |  |
| **Pluripotency Genes** | | | |
| Endo-OCT4 | FP | 5'-AGTTTGTGCCAGGGTTTTTG-3’ | NM_001285987.1 |
|  | RP | 5'-ACTTCACCTTCCCTCCAACC-3’ |  |
| Endo-SOX2 | FP | 5'-GGGAAATGGGAGGGGTGCAAAAGAGG-3’ | NM_003106.3 |
|  | RP | 5'-TTGCGTGAGTGTGGATGGGATTGGTG  -3’ |  |
| NANOG | FP | 5'-CTCTCCAACATCCTGAACCTC-3’ | NM_001297698 |
|  | RP | 5'-ACACCATTGCTATTCTTCGG-3’ |  |
| c-MYC | FP | 5'-GGATTCTCTGCTCTCCTCGAC-3’ | NM_002467.5 |
|  | RP | 5'-CTTCCTCATCTTCTTGTTCCT-3’ |  |
| KLF-4 | FP | 5'-AAACCTACACAAAGAGTTCCCAT-3’ | NP_001300981.1 |
|  | RP | 5'-TTTCCATCCACAGCCGTCCCA-3’ |  |
| REX-1 | FP | 5'-AACAGGATGAAGCAGATTAACAGA-3’ | NM_001304358.1 |
|  | RP | 5'-TGTAGGAGCATCTTAGTAACACC-3’ |  |
| DNMT3a | FP | 5'-CCTCAAACCCAACAACACGCAAC-3’ | NM_001320893.1 |
|  | RP | 5'-TCTGATCTTCATCCCCTCGGTCT-3’ |  |
| UTF-1 | FP | 5'-AGTTCCTTAAAGACAAGTTTCGC-3’ | NM_003577.2 |
|  | RP | 5'-CAGCAGCCCCATGAGCTTCC-3’ |  |
| CDH-1 | FP | 5'-ACCTTCCTCCCAATACATCTCC-3’ | NM_004360.4 |
|  | RP | 5'-TTGTAGTCACCCACCTCTAAGGC-3’ |  |
| **Episomal Plasmid Markers** | | | |
| OriP | FP | 5'-TTCCACGAGGGTAGTGAA CC-3' | ThermoFisher Epi5™ Episomal iPSC Reprogramming Kit- A15960 |
|  | RP | 5'-TCGGGGGTGTTAGAGACAAC-3' |  |
| **Sendai Viral Genome Markers** | | | |
| SeV | FP | 5'-GGATCACTAGGTGATATCGAGC-3’ | Thermo Fisher CytoTune-iPS Sendai Reprogramming- A16517 |
|  | RP | 5'-ACCAGACAAGAGTTTAAGAGATATGTATC-3’ |  |
